# A blood microRNA classifier for the prediction of ICU mortality in COVID-19 patients: a multicenter validation study

**DOI:** 10.1186/s12931-023-02462-x

**Published:** 2023-06-17

**Authors:** David de Gonzalo-Calvo, Marta Molinero, Iván D. Benítez, Manel Perez-Pons, Nadia García-Mateo, Alicia Ortega, Tamara Postigo, María C. García-Hidalgo, Thalia Belmonte, Carlos Rodríguez-Muñoz, Jessica González, Gerard Torres, Clara Gort-Paniello, Anna Moncusí-Moix, Ángel Estella, Luis Tamayo Lomas, Amalia Martínez de la Gándara, Lorenzo Socias, Yhivian Peñasco, Maria Del Carmen de la Torre, Elena Bustamante-Munguira, Elena Gallego Curto, Ignacio Martínez Varela, María Cruz Martin Delgado, Pablo Vidal-Cortés, Juan López Messa, Felipe Pérez-García, Jesús Caballero, José M. Añón, Ana Loza-Vázquez, Nieves Carbonell, Judith Marin-Corral, Ruth Noemí Jorge García, Carmen Barberà, Adrián Ceccato, Laia Fernández-Barat, Ricard Ferrer, Dario Garcia-Gasulla, Jose Ángel Lorente-Balanza, Rosario Menéndez, Ana Motos, Oscar Peñuelas, Jordi Riera, Jesús F. Bermejo-Martin, Antoni Torres, Ferran Barbé

**Affiliations:** 1 Translational Research in Respiratory Medicine, University Hospital Arnau de Vilanova and Santa Maria, IRBLleida, Lleida, Spain; 2 CIBER of Respiratory Diseases (CIBERES), Institute of Health Carlos III, Madrid, Spain; 3 Group for Biomedical Research in Sepsis (BioSepsis), Instituto de Investigación Biomédica de Salamanca, (IBSAL), Gerencia Regional de Salud de Castilla y León, Salamanca, Spain; 4 Department of Medicine, Intensive Care Unit University Hospital of Jerez, University of Cádiz, INIBiCA, Cádiz Spain; 5 Critical Care Department, Hospital Universitario Río Hortega de Valladolid, Valladolid, Spain; 6 Department of Intensive Medicine, Hospital Universitario Infanta Leonor, Madrid, Spain; 7 Intensive Care Unit, Hospital Son Llàtzer, Palma de Mallorca, Illes Balears, Spain; 8 Servicio de Medicina Intensiva, Hospital Universitario Marqués de Valdecilla, Santander, Spain; 9 Servei de Medicina Intensiva, Hospital de Mataró (Consorci Sanitari del Maresme), Mataró, Spain; 10 Department of Intensive Care Medicine, Hospital Clínico Universitario Valladolid, Valladolid, Spain; 11 Unidad de Cuidados Intensivos, Hospital Universitario San Pedro de Alcántara, Cáceres, Spain; 12 Critical Care Department, Hospital Universitario Lucus Augusti, Lugo, Spain; 13 Hospital Universitario Torrejón-Universidad Francisco de Vitoria, Madrid, Spain; 14 Intensive Care Unit, Complexo Hospitalario Universitario de Ourense, Ourense, Spain; 15 Complejo Asistencial Universitario de Palencia, Palencia, Spain; 16 Servicio de Microbiología Clínica, Facultad de Medicina, Departamento de Biomedicina y Biotecnología, Hospital Universitario Príncipe de Asturias – Universidad de Alcalá, Madrid, Spain; 17 Centro de Investigación Biomédica en Red en Enfermedades Infecciosas (CIBERINFEC), Instituto de Salud Carlos III, Madrid, Spain; 18 Grup de Recerca Medicina Intensiva, Intensive Care Department Hospital, Universitari Arnau de Vilanova, Lleida, Spain; 19 Servicio de Medicina Intensiva. Hospital Universitario La Paz, IdiPAZ, Madrid, Spain; 20 Unidad de Medicina Intensiva, Hospital Universitario Virgen de Valme, Seville, Spain; 21 Intensive Care Unit, Hospital Clínico y Universitario de Valencia, Valencia, Spain; 22 Critical Care Department, Hospital del Mar-IMIM, Barcelona, Spain; 23 Intensive Care Department, Hospital Nuestra Señora de Gracia, Zaragoza, Spain; 24 Intensive Care Department, University Hospital Santa María, IRBLleida, Lleida, Spain; 25 Servei de Pneumologia, Hospital Clinic, Universitat de Barcelona, IDIBAPS, Barcelona, Spain; 26 Intensive Care Department, SODIR Research Group, Vall d’Hebron Hospital Universitari, Vall d’Hebron Institut de Recerca (VHIR), Barcelona, Spain; 27 Barcelona Supercomputing Center (BSC), Barcelona, Spain; 28 Hospital Universitario de Getafe, Madrid, Spain; 29 Dep. of Medicine, Universidad Europea, Madrid, Spain; 30 Dep. of Bioengineering, Universidad Carlos III, Madrid, Spain; 31 Pulmonology Service, University and Polytechnic Hospital La Fe, Valencia, Spain; 32 Hospital Universitario Río Hortega de Valladolid, Valladolid, Spain; 33 Institució Catalana de Recerca i Estudis Avançats (ICREA), Barcelona, Spain

**Keywords:** Biomarker, COVID-19, ICU, microRNA, Prognosis, SARS-CoV-2

## Abstract

**Background:**

The identification of critically ill COVID-19 patients at risk of fatal outcomes remains a challenge. Here, we first validated candidate microRNAs (miRNAs) as biomarkers for clinical decision-making in critically ill patients. Second, we constructed a blood miRNA classifier for the early prediction of adverse outcomes in the ICU.

**Methods:**

This was a multicenter, observational and retrospective/prospective study including 503 critically ill patients admitted to the ICU from 19 hospitals. qPCR assays were performed in plasma samples collected within the first 48 h upon admission. A 16-miRNA panel was designed based on recently published data from our group.

**Results:**

Nine miRNAs were validated as biomarkers of all-cause in-ICU mortality in the independent cohort of critically ill patients (FDR < 0.05). Cox regression analysis revealed that low expression levels of eight miRNAs were associated with a higher risk of death (HR from 1.56 to 2.61). LASSO regression for variable selection was used to construct a miRNA classifier. A 4-blood miRNA signature composed of miR-16-5p, miR-192-5p, miR-323a-3p and miR-451a predicts the risk of all-cause in-ICU mortality (HR 2.5). Kaplan‒Meier analysis confirmed these findings. The miRNA signature provides a significant increase in the prognostic capacity of conventional scores, APACHE-II (C-index 0.71, DeLong test p-value 0.055) and SOFA (C-index 0.67, DeLong test p-value 0.001), and a risk model based on clinical predictors (C-index 0.74, DeLong test-p-value 0.035). For 28-day and 90-day mortality, the classifier also improved the prognostic value of APACHE-II, SOFA and the clinical model. The association between the classifier and mortality persisted even after multivariable adjustment. The functional analysis reported biological pathways involved in SARS-CoV infection and inflammatory, fibrotic and transcriptional pathways.

**Conclusions:**

A blood miRNA classifier improves the early prediction of fatal outcomes in critically ill COVID-19 patients.

**Supplementary Information:**

The online version contains supplementary material available at 10.1186/s12931-023-02462-x.

## Background

The COVID-19 pandemic has exerted dramatic pressure on the health care system globally, especially in intensive care units (ICUs). COVID-19 is characterized by substantial heterogeneity in progression rates [1], posing a considerable challenge to triage critically ill patients, inform early intervention and guide both ICU capacity and resource allocation. In this scenario, reliable biomarkers would be invaluable to improve risk stratification and allow for more effective clinical decision-making. These biomarkers may also be legitimate targets for therapeutic intervention to prevent ICU complications and adverse outcomes.

MicroRNAs (miRNAs) are small noncoding RNAs involved in posttranscriptional gene regulation that have emerged as innovative biomarkers for a number of conditions [2–4], including viral respiratory infections [5] and the management of critical patients [6]. Circulating miRNAs are cost-effective biomarkers that can be quantified through a minimally invasive blood draw using techniques already employed in clinical laboratories [7]. Furthermore, recent clinical trials have demonstrated that miRNA-based therapies seem to be well tolerated and show promising effects [8].

Here, we used samples from a large multicenter cohort of critically ill patients with COVID-19, i.e., the CIBERESUCICOVID study (NCT04457505), to construct a blood miRNA classifier that could be used to predict all-cause in-ICU mortality. We focused our attention on a 16-miRNA panel previously associated by our group with the severity of the disease and its adverse clinical outcomes in a miRNA biomarker discovery study [9]. To the best of our knowledge, our study is the largest on miRNAs as biomarkers for the clinical management of critically ill COVID-19 patients.

## Patients and methods

### Study design and data collection

This is a substudy of the CIBERESUCICOVID study registered at www.clinicaltrials.gov with the identification NCT04457505. CIBERESUCICOVID is a multicenter, observational, prospective/retrospective cohort study that enrolled critically ill COVID-19 patients admitted to the ICUs of 55 Spanish hospitals [10]. CIBERESUCICOVID started in May 2020 by collecting the retrospective data of patients admitted to participating ICUs (from February 2020) and continued prospectively until February 2021. After enrollment, comprehensive demographic, clinical, pharmacological and laboratory data were exhaustively collected at hospital and ICU admission, as previously described in Torres et al. [10]. The pharmacologic treatments administered and interventions performed during hospital admission until either discharge from hospital or death were also collected. Definitions have also been previously published [11]. Deidentified patient data were abstracted manually from the electronic medical records and stored in a Research Electronic Data Capture (REDCap) database hosted in the Centro de Investigación Biomédica en Red (CIBER, Madrid, Spain). Data from patients’ medical records were incorporated into the database by trained local researchers. The study coordinators ensured the integrity and timely completion of data collection. Prior to statistical analyses, incoherent or missing data were checked by independent experienced data collectors trained in critical care.

The study protocol was approved by the respective ethics committee of each participating hospital. The study was designed and conducted in compliance with the Declaration of Helsinki and national and international law on data protection. Participants, or their legal representatives, provided informed consent, when possible, for the use of the samples and data. In the remaining cases, an informed consent waiver was authorized by the ethics committee.

### Primary and secondary outcomes

The primary outcome was all-cause in-ICU mortality. Secondary outcomes included all-cause 28-day mortality and all-cause 90-day mortality from ICU admission and length of hospital stay, ICU stay and invasive mechanical ventilation (IMV).

### Study sample

Patients admitted to the ICU at the participating hospitals were enrolled in the current substudy if they fulfilled the following inclusion criteria: age over 18, laboratory-confirmed SARS-CoV-2 infection according to a standardized test, admission to the ICU and blood sample collected during the first 48 h available. Patients were excluded if they had unconfirmed SARS-CoV-2 infection, lacked data at baseline or hospital discharge or were admitted to an ICU for other causes. A list of participating hospitals is provided in Supplemental Figure S1 (Additional File 2).

### Sample size calculation

The sample size calculation was based on comparisons of miRNA levels between study groups (survivors vs. nonsurvivors) using a two-sample t test. Relevant biological differences were considered as a fold change of 1.2 (or 0.83 in downregulated miRNAs). According to our previous data [9], we assumed a coefficient of variation of 0.5. The significance level was fixed at 0.05. A minimum sample size of 143 per group was necessary to achieve 90% statistical power. Expecting a mortality rate of 33%, a total sample size of 429 samples was necessary for the validation study. The sample size used was higher to ensure the necessary number of patients. Ultimately, 503 patients from the CIBERESUCICOVID consortium with blood samples available were included.

### Experimental methods

Details regarding miRNA quantification and prediction of miRNA target regulation are provided in the Supplemental Methods (Additional File 1).

### Statistical analysis

The characteristics of the study population were summarized by descriptive statistics. Data are presented as the medians [P_25_; P_75_] for continuous variables and as frequencies (percentage) for categorical variables. Continuous variables were compared using the Mann‒Whitney U test. Categorical variables were compared using the Fisher’s exact test. Linear models with Empirical Bayes statistic were used to evaluate differences in miRNA levels between survivors and nonsurvivors [12]. miRNAs with a significant difference [false discovery rate (FDR) < 0.05] between study groups and a fold change (FC) higher than 1.2 (or lower than 0.83 for downregulated levels) were considered differentially detected. Adjusted models were evaluated, including potential confounding factors that could affect the association of the miRNA and the outcome. The Pearson correlation coefficient was used to assess the correlation between continuous variables. Correlations between validated miRNAs and laboratory parameters were performed in the whole population. Correlations between the miRNAs included in the 4-blood miRNA classifier and length of hospital stay, ICU stay or IMV were performed in survivors of ICU stay. A blood miRNA classifier for all-cause in-ICU mortality was constructed using a relaxed least absolute shrinkage and selection operator (LASSO) model. This approach was used to reduce the collinearity of the multivariable model due the high correlation between the plasma levels of miRNAs. Fivefold cross-validation was carried out to determine the lambda parameter of the LASSO model. Lambda and gamma parameters were selected as the values associated with one standard error greater than the minimum mean square error (MSE). The miRNA levels were standardized prior to fitting the LASSO model. For levels of selected miRNAs, a cutoff point was established for fitted mortality risk using a maximally selected log-rank statistic [13]. For the blood miRNA classifier, in order to improve the clinical interpretation, patients were classified in high- or low-risk based on their probability of mortality, estimated by the final model, using the same method. The hazard ratio (HR) was estimated using Cox regression models including the dichotomized levels of individual miRNAs and the blood miRNA classifier (high- and low-risk groups) [13]. Kaplan‒Meier curves were used to illustrate differences among groups in the time-to-event outcome, and the log-rank test was performed to assess statistical significance. The clinical model for fatal outcomes included demographic characteristics, baseline comorbidities, laboratory tests, organ support and oxygenation variables: age (years), sex (female vs. male), hypertension (yes vs. no), chronic respiratory disease (yes vs. no), chronic kidney disease (yes vs. no), diabetes (yes vs. no) and at ICU admission: PaO_2_/FiO_2_ ratio, pH, lymphocyte count (x10^9^/L), platelet count (x10^9^/L), D-dimer (ng/mL), urea (mg/dL) and serum creatinine (mg/dL). The variables were included based on clinical relevance and bibliography [11]. Logistic regression models were used to analyze the association between the miRNA classifier and 28-day and 90-day mortality. The C-index and area under the ROC curve (AUC) were calculated for survival and logistic models, respectively. The incremental gain in model discrimination was evaluated using DeLong’s test. The p-value threshold defining statistical significance was set at < 0.05. All statistical analyses were performed using R software, version 4.0.2.

## Results

### Study sample characteristics

The main baseline characteristics of the study cohort are summarized in Table 1. The substudy included patients admitted from March 2020 to February 2021. The median age was 65.0 [56.0;73.0] years, and 31.6% were females. Of the total subcohort, 29.3% died during the ICU stay. The 28-day and 90-day mortality rates were 21.4% and 28.9%, respectively. Nonsurvivors were older and had a higher prevalence of comorbidities, including hypertension, diabetes mellitus and chronic kidney disease. At ICU admission, this group had significantly higher APACHE-II and SOFA scores. At the same time, nonsurvivors had a lower PaO_2_/FiO_2_ and a higher PaCO_2_ than survivors. Laboratory parameters highlighted disparities between survivors and nonsurvivors in neutrophil counts, lymphocyte counts, creatinine, urea and D-dimer. Patients who died during the ICU stay required a higher use of IMV and prone positioning.

**Table 1:** Characteristics of study sample

	ALL	Survivor	Nonsurvivor	p-value	Available data
	N = 491	N = 347	N = 144		
**Sociodemographic characteristics**					
Age (years), median [P25; P75]	65.0 [56.0;73.0]	62.0 [53.0;70.0]	71.5 [62.8;76.0]	< 0.001	491
Female, n (%)	155 (31.6%)	107 (30.9%)	48 (33.3%)	0.678	490
Body mass index (kg/cm^2^), median [P25; P75]	29.0 [26.1;32.7]	29.0 [26.1;33.5]	28.8 [26.0;31.1]	0.156	410
Smoking history, n (%)				0.676	472
Former	145 (30.7%)	100 (30.0%)	45 (32.4%)		
Nonsmoker	289 (61.2%)	204 (61.3%)	85 (61.2%)		
Current	38 (8.05%)	29 (8.71%)	9 (6.47%)		
**Comorbidities**					
Hypertension, n (%)	275 (56.0%)	179 (51.6%)	96 (66.7%)	0.003	491
Diabetes Mellitus, n (%)	137 (27.9%)	83 (23.9%)	54 (37.5%)	0.003	491
Obesity, n (%)	184 (37.5%)	146 (42.1%)	38 (26.4%)	0.002	491
Chronic cardiovascular disease, n (%)	66 (13.5%)	43 (12.4%)	23 (16.0%)	0.367	490
Chronic pulmonary disease, n (%)	59 (12.0%)	37 (10.7%)	22 (15.3%)	0.201	491
Chronic kidney disease, n (%)	40 (8.16%)	23 (6.63%)	17 (11.9%)	0.080	490
**Disease chronology**					
Time since first symptoms to ICU admission (days), median [P25; P75]	9.00 [7.00;12.0]	9.00 [7.00;12.0]	8.00 [6.00;11.2]	0.167	491
Time since hospital admission to ICU admission (days), median [P25; P75]	2.00 [0.00;4.00]	2.00 [0.00;3.00]	2.00 [0.00;4.25]	0.449	491
Hospital stay (days), median [P25; P75]	25.0 [15.0;41.5]	27.0 [16.0;45.5]	22.0 [14.8;32.2]	0.029	491
ICU stay (days), median [P25; P75]	14.0 [7.00;27.5]	12.0 [6.00;27.0]	19.0 [11.8;28.0]	< 0.001	491
**Blood gases and laboratory parameters at ICU admission**					
Oxygen saturation (%), median [P25; P75]	94.0 [90.0;97.0]	94.8 [91.5;97.0]	92.3 [87.0;96.0]	< 0.001	483
PaCO_2_ (mmHg), median [P25; P75]	38.7 [33.2;45.0]	37.0 [33.0;45.0]	41.0 [34.3;50.6]	0.002	464
pH, median [P25; P75]	7.41 [7.35;7.45]	7.43 [7.37;7.46]	7.38 [7.28;7.44]	< 0.001	465
PaO_2_/FiO_2_, median [P25; P75]	107 [76.0;148]	109 [81.8;153]	100 [65.6;136]	0.008	449
Glucose (mg/dL), median [P25; P75]	159 [129;207]	154 [126;197]	174 [135;255]	0.001	486
Creatinine (mg/dL), median [P25; P75]	0.84 [0.70;1.15]	0.82 [0.69;1.04]	0.90 [0.70;1.40]	0.035	487
C-reactive protein (mg/L), median [P25; P75]	121 [57.0;216]	114 [56.8;199]	150 [59.4;234]	0.147	472
D-dimer (ng/mL), median [P25; P75]	1023 [562;1898]	836 [484;1536]	1578 [976;3822]	< 0.001	451
Leukocyte count (x10^9^/L), median [P25; P75]	9.90 [7.20;13.8]	9.49 [6.95;13.0]	11.7 [8.06;15.8]	< 0.001	487
Neutrophil count (x10^9^/L), median [P25; P75]	8.90 [6.17;12.5]	8.30 [5.80;11.4]	10.6 [7.30;14.8]	< 0.001	468
Lymphocyte count (x10^9^/L), median [P25; P75]	0.63 [0.41;0.88]	0.68 [0.50;0.90]	0.54 [0.35;0.80]	< 0.001	477
Monocyte count (x10^9^/L), median [P25; P75]	0.40 [0.23;0.56]	0.40 [0.25;0.56]	0.34 [0.20;0.58]	0.179	465
Platelet count (x10^9^/L), median [P25; P75]	236 [185;307]	237 [190;304]	230 [165;317]	0.131	485
AST (U/L), median [P25; P75]	42.6 [29.0;68.0]	42.0 [29.0;68.0]	42.8 [29.0;68.3]	0.840	419
ALT (U/L), median [P25; P75]	41.0 [25.0;67.0]	42.5 [27.0;67.8]	35.5 [21.2;63.0]	0.019	448
Urea (mg/dL), median [P25; P75]	52.0 [38.0;73.0]	49.0 [35.0;68.0]	62.2 [46.2;94.0]	< 0.001	445
**Severity scores at ICU admission**					
APACHE-II score, median [P25; P75]	12.0 [9.00;17.0]	11.0 [8.00;15.0]	15.0 [12.0;21.2]	< 0.001	356
SOFA Score, median [P25; P75]	5.00 [4.00;7.25]	5.00 [4.00;7.00]	7.00 [4.00;8.75]	< 0.001	380
**Interventions during ICU stay**					
Antibiotics, n (%)	461 (94.1%)	321 (92.5%)	140 (97.9%)	0.037	490
Hydroxychloroquine, n (%)	32 (6.54%)	26 (7.49%)	6 (4.23%)	0.261	489
Tocilizumab, n (%)	125 (25.6%)	92 (26.5%)	33 (23.2%)	0.523	489
Corticoids, n (%)	483 (98.8%)	341 (98.6%)	142 (99.3%)	0.676	489
High flow oxygen nasal cannula, n (%)	347 (80.5%)	265 (84.9%)	82 (68.9%)	< 0.001	431
Noninvasive positive pressure ventilation, n (%)	144 (29.9%)	96 (28.2%)	48 (34.3%)	0.221	481
Invasive mechanical ventilation, n (%)	384 (78.4%)	247 (71.4%)	137 (95.1%)	< 0.001	490
Prone positioning, n (%)	277 (56.5%)	161 (46.4%)	116 (81.1%)	< 0.001	490

*ALT: alanine aminotransferase; AST: aspartate aminotransferase; FiO*_*2*_: *fraction of inspired oxygen; ICU: Intensive care unit; PaCO*_*2*_: *carbon dioxide partial pressure; PaO*_*2*_: *oxygen partial pressure*

### Differential microRNA profiles among ICU survivors and nonsurvivors: validation study

**Fig. 1:**
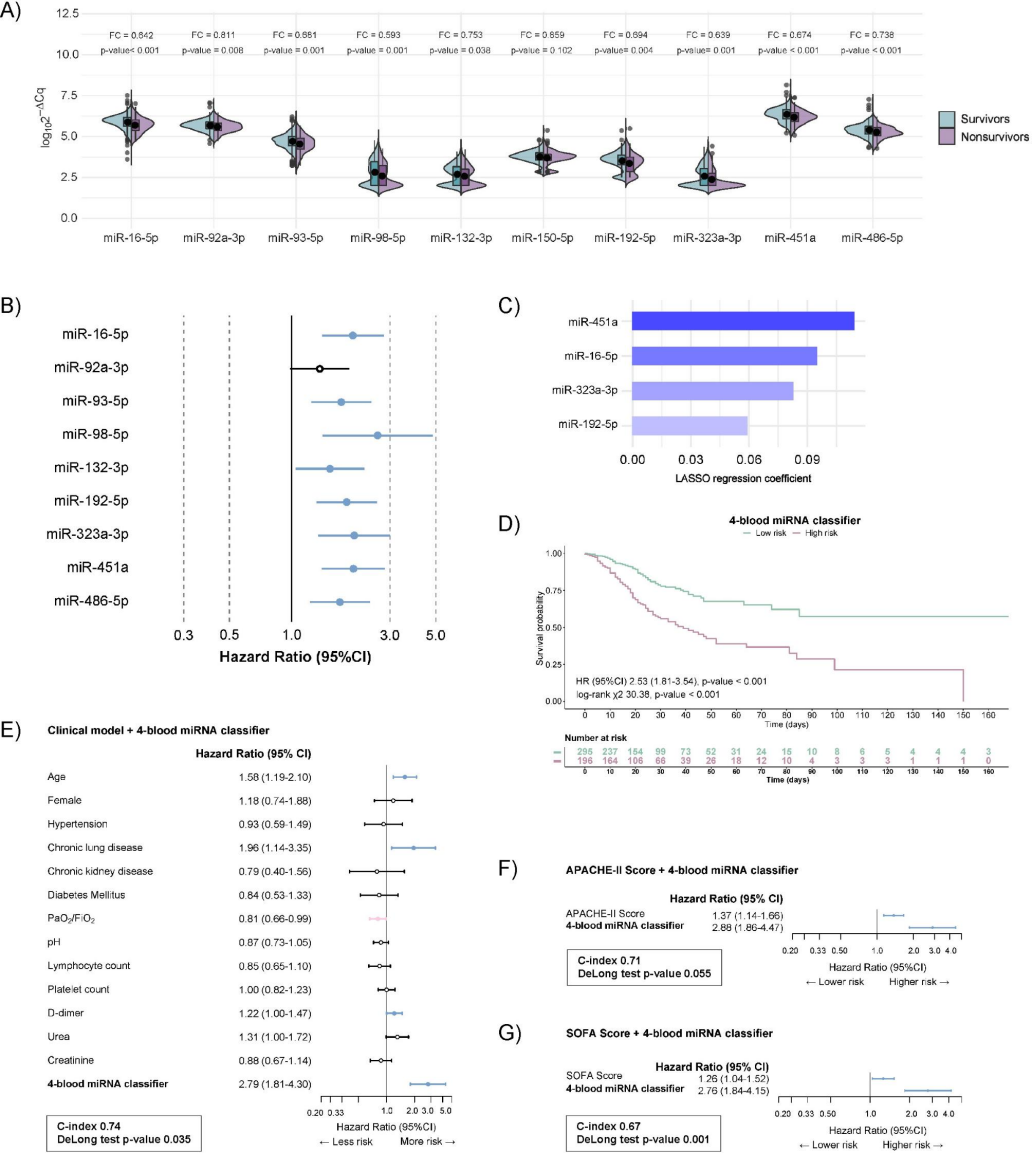
**Construction of the 4-blood microRNA classifier.** (**A**) Violin plot comparing validated microRNA levels between survivors and nonsurvivors to ICU stay. Between-group differences were analyzed using linear models for arrays. P-values describe the significance level for each comparison; (**B**) hazard ratio (HR) and 95% confidence interval (90% CI) for each validated microRNA. Cox regression models include the dichotomized levels of individual miRNAs; (**C**) Blood microRNA classifier constructed using a selection process based on relaxed least absolute shrinkage and selection operator (LASSO) model; (**D**) Kaplan‒Meier estimations for the 4-blood microRNA classifier. (**E-G**) Combination of the 4-blood microRNA classifier with established clinical predictors or contemporaneous prognostic scores. (**E**) Clinical model (n = 373); (**F**) APACHE-II (n = 352); (**G**) SOFA (n = 379). The graph displays the hazard ratio (HR) and 95% confidence interval (90% CI) for each variable. Cox regression models included the dichotomized levels of the 4-blood microRNA classifier. The hazard ratio (95% CI) is displayed as a 1-SD change for continuous predictors

First, we interrogated our candidate miRNAs in an independent cohort of critically ill COVID-19 patients (Supplemental Table S1; Additional File 2). Linear models with the empirical Bayes statistic were used to identify differentially expressed miRNAs between survivors and nonsurvivors. From the sixteen candidates, six miRNAs (miR-27a-3p, miR-27b-3p, miR-148a-3p, miR-199a-5p, miR-214-3p and miR-491-5p) showed contradictory results when compared with previous findings (Supplemental Figure S2; Additional File 2). Furthermore, the previous association between miR-150-5p and COVID-19 was not observed in the current study (FDR 0.102) (Fig. 1A). Therefore, these seven miRNAs were discarded from subsequent analysis. Ultimately, nine miRNAs reached a statistically significant signal (FC < 0.83, FDR < 0.05) and showed similar results to those in the previous study in terms of the size effect and direction of the association: miR-16-5p, miR-92a-3p, miR-93-5p, miR-98-5p, miR-132-3p miR-192-5p, miR-323a-3p, miR-451a and miR-486-5p (Fig. 1A). No great impact of confounding factors was observed in the association between miRNA levels and all-cause in-ICU mortality (Supplemental Table S2; Additional File 2). Poor correlations were observed between validated miRNAs and laboratory parameters (rho < 0.3) (Supplemental Figure S3; Additional File 2). The nine validated miRNAs entered the next phase.

### A blood microRNA classifier for predicting ICU mortality risk in critically ill COVID-19 patients

Cox regression analysis revealed that low expression of eight miRNAs predicted the highest risk of all-cause in-ICU mortality (HR from 1.54 to 2.61) (Fig. 1B & Supplemental Table S3; Additional File 2). No association was observed for miR-92a-3p [HR 1.37, p-value 0.06]. We next constructed a blood miRNA classifier of all-cause in-ICU mortality using the LASSO algorithm for feature selection. A 4-blood miRNA signature consisting of miR-16-5p, miR-192-5p, miR-323a-3p and miR-451a was selected (Fig. 1C). Using the classifier, the group of patients at higher risk of in-ICU mortality showed an HR (95% CI) of 2.53 (1.81–3.54) compared to the low-risk group. The Kaplan–Meier curve revealed clear separation of survival between the high- and low-risk subgroups (log-rank χ2 30.38, p-value < 0.001) (Fig. 1D). The 4-blood miRNA classifier was also significantly associated with 28-day mortality [OR (95% CI) 3.50 (2.34–5.29), p-value < 0.001] and 90-day mortality [OR (95% CI) 3.65 (2.44–5.51), p-value < 0.001]. No significant correlations were found between the classifier and the length of hospital stay, ICU stay or IMV in critically ill survivors (Supplemental Figure S4; Additional File 2).

Then, we explored whether the 4-blood miRNA classifier cooperatively discriminates all-cause in-ICU mortality in combination with established clinical predictors or contemporaneous prognostic scores. To this end, we combined a model based on clinical predictors and the prognostic scores APACHE-II and SOFA with the 4-blood miRNA classifier (Fig. 1E & 1F & 1G). The addition of the miRNA signature to available clinical parameters improved risk discrimination: miRNA classifier-clinical model pair: C-index 0.74, DeLong test p-value 0.035; miRNA classifier-APACHE-II pair: C-index 0.71, DeLong test p-value 0.055; and miRNA classifier-SOFA pair: C-index 0.67, DeLong test p-value 0.001. Concerning 28-day and 90-day mortality, the addition of the miRNA classifier also improved the prognostic capacity of the clinical model, APACHE-II and SOFA (Supplemental Figure S5; Additional File 2).

Of note, the ability of the 4-blood miRNA classifier to predict mortality risk remained significant after extensive adjustment for clinical predictors, APACHE-II or SOFA (Fig. 1E & 1F & 1G). Indeed, we analyzed the relationship between the 4-blood miRNA classifier and established clinical features of the disease, including clinically available blood biomarkers. Again, poor correlations (rho < 0.3) were observed between the miRNA score with blood cell counts and biochemical parameters (Supplemental Figure S6; Additional File 2).

### Functional analysis of the 4-blood microRNA classifier

**Fig. 2:**
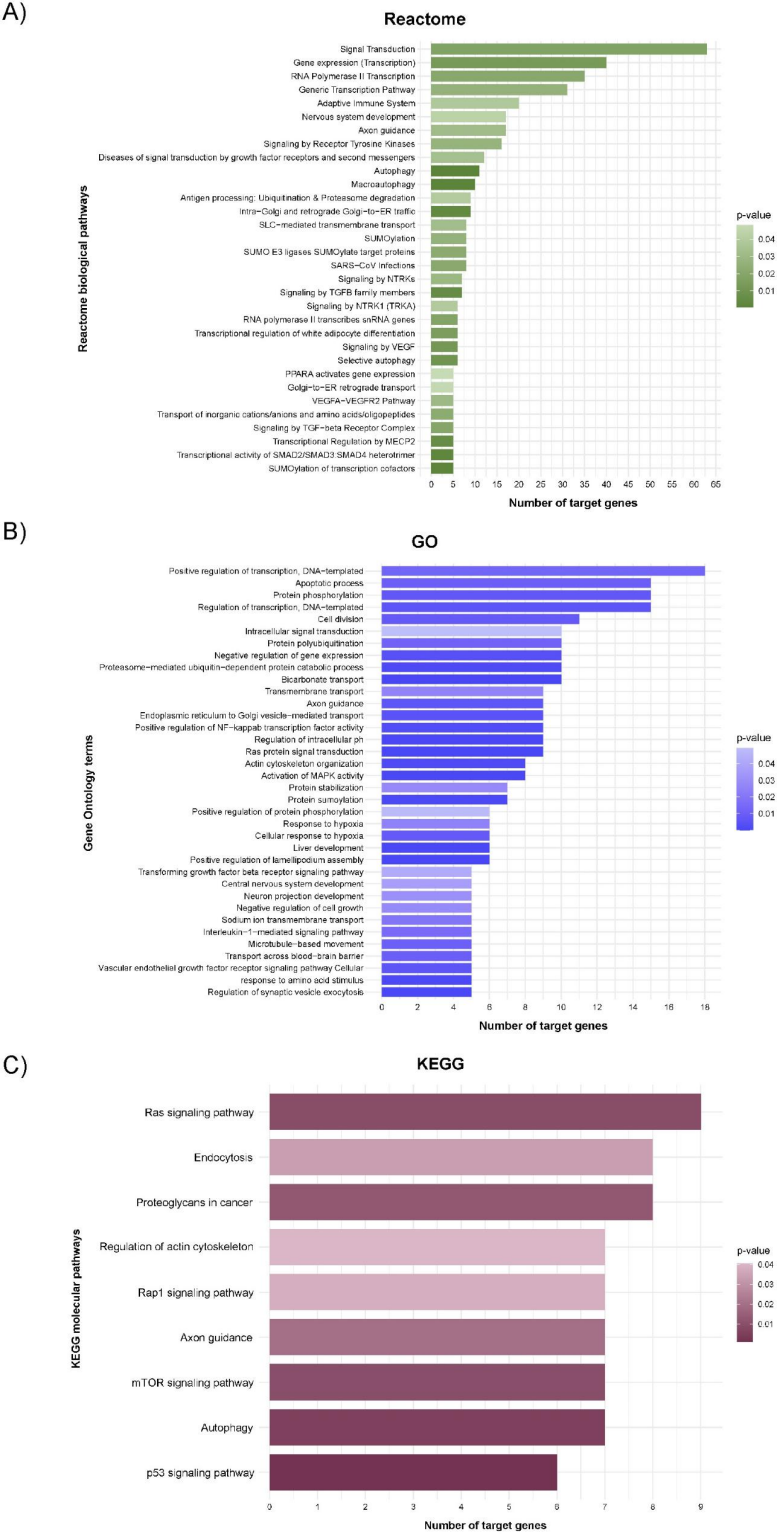
**Functional enrichment analysis of the 4-blood microRNA signature.** The microRNAs that composed the classifier were included in the analyses. Graph representing the p-value versus the number of target genes for each microRNA using the Reactome (A), GO (B) and KEGG (C) databases. miRWalk2.0 (accessed date July 26th, 2022) was used to predict the interaction of microRNAs with their targets (TargetScan filter)

The components of the classifier were subjected to Reactome, GO and KEGG analyses. The functional analysis reported 32 Reactome biological pathways, 36 GO terms and 9 KEGG molecular pathways. We identified downstream targets and mechanisms associated with viral infections, such as pathways implicated in transcriptional regulation and SARS-CoV infections, and processes implicated in COVID-19 physiopathology, including the NF-κB, VEGF and TGF signaling pathways. (Fig. 2).

## Discussion

The actual prognosis of ICU mortality in COVID-19 patients constitutes a challenge because of the lack of risk assessment metrics [1]. Determining early predictors of mortality is mandatory to guide ICU capacity and resource allocation. To identify novel biomarkers, the plasma of critically ill COVID-19 patients obtained within the first 48 h of ICU admission was subjected to miRNA profiling.

In the current study, we first validated the potential use of nine miRNA candidates selected from a previous discovery study as advanced mortality predictors in the ICU. Our differentially expressed miRNAs quantified in an independent multicenter cohort show a high concordance with our own previous data [9]. Then, we explored the predictive potential of the validated candidates. Accordingly, we showed that eight miRNAs were associated with in-ICU survival. Furthermore, we constructed a 4-blood miRNA classifier that provides an innovative estimate of early in-ICU mortality prediction. The association between the classifier and mortality persisted even after multivariable adjustment that included clinical history and established biomarkers. In this sense, neither individual components of the 4-blood miRNA classifier nor the signature were correlated with prominent features of SARS-CoV-2 infection and disease severity, such as leukocyte counts, including neutrophil and lymphocyte counts, D-dimer or creatinine, among others, suggesting that the miRNA classifier may provide novel and independent information for prognostication. No correlation was observed between the classifier and length of hospital stay, ICU stay or IMV in critically ill survivors. The explanation of this result is unclear. It may be associated with differences in the study sample or the pathobiological mechanisms implicated in the different clinical outcomes. Finally, we demonstrated that the use of the 4-blood miRNA classifier in concert with conventional scores of adverse outcomes, i.e., APACHE-II and SOFA, and a model based on clinical predictors is more accurate than either alone for the prognostication of fatal outcomes. Indeed, the combination of the host miRNA classifier with the clinical predictors computes prognosis for all-cause in-ICU, 28-day and 90-day mortality more precisely than any information currently available at ICU admission.

In addition, to support the previous association reported by our group between miR-16-5p, miR-98-5p, miR-132-3p, miR-192-5p and miR-323a-3p and in-ICU mortality [9], these results are in line with findings from the literature. For instance, Fernández-Pato et al. recently demonstrated that plasma miR-98-5p is reduced in severe COVID-19 patients [14]. In a study from Wilson et al., both miR-323-3p and miR-451a were shown to be downregulated in plasma samples from individuals with severe disease [15]. Overall, our miRNA signature introduces a new horizon for a combinatorial clinical data–transcriptomic biomarker systems for mortality prognostication in critically ill COVID-19 populations. This advancement is especially relevant since only a few potential blood-based biomarkers, mainly inflammatory and thrombotic mediators [16–18], have been proposed for the prognostication of critically ill COVID-19 patients. Furthermore, recent findings suggest the superior specificity of miRNA for COVID-19 mortality compared to protein biomarkers [19]. Risk stratification based on progression rate can optimize triage, inform early intervention, improve the allocation of hospital resources and allow patient selection for clinical trials. Additional work will be necessary to determine whether the integration of electronic health record data with the host miRNA classifier, which can be quantified with relatively low cost through techniques already available in clinical laboratories such as qPCR, is suitable for use as a prognostic test in the ICU.

The development of novel therapies to improve outcomes in critically ill COVID-19 patients remains a challenge [20]. Rational therapeutic approaches could be inferred from the current results. Disease- and pathway-specific biomarkers that can predict patients’ evolution in the ICU may also constitute therapeutic targets. In this context, a second notable finding from our study is that the functional analysis of the miRNA classifier components has identified relative enrichment of pathways not only implicated in inflammatory mechanisms but also in VEGF, TGF and transcriptional regulation signaling, which are hallmarks of viral infection and disease severity [14, 21, 22]. Notably, a biological pathway related to SARS-CoV infection was enriched in the targets of the miRNA signature. The biological function reported in independent miRNA-based studies was also captured in our analysis. miR-16-5p and miR-98-5p have been predicted to target the SARS-CoV-2 genome and host factors that mediate viral infectivity [23, 24]. Interestingly, miR-16-5p is downregulated in macrophages exposed to SARS-CoV‐2 virion spike 1 glycoprotein (S1) [25]. The levels of the miRNA are upregulated in response to pro‐resolving mediators, which suggests a role of miR-16-5p in the resolution of inflammation and the return to homeostasis after viral stimuli. The downregulation of miR-451a levels has been described as a possible mechanism implicated in cytokine storms in COVID-19 patients [26]. Therefore, the association of these miRNAs as biomarkers for fatal outcomes may not be coincidental. Potential miRNA-based antiviral therapeutics warrant consideration [27].

### Strengths and limitations

The strengths of the study are the use of a multicenter design including patients from 19 hospitals from different geographical locations, the use of *a priori* sample size calculation, the high number of patients analyzed, the rigorous control of data quality and the evaluation of the miRNA classifier in conjunction with electronic health history and contemporaneous tests. The study population is a subcohort of critically ill COVID-19 patients collected in the same time frame as the nationwide cohort CIBERESUCICOVID [10]; and therefore, it has similar characteristics being females (29.6%), with a median age of 63 [54–71] and hypertension (49.7%), obesity (36.3%) and diabetes mellitus (24.6%) as the most common comorbidities (Supplemental Table S4; Additional File 2). In addition, the subcohort is similar to populations described in other studies, such as Dongelmans et al. [28] and Carbonell et al. [29]. The comparison of the biomarker performance with clinical predictors is also fundamental to obtaining robust evidence for the potential clinical applicability of the miRNA classifier. The low number of miRNAs constitutes an advantage for its potential translation to patient management.

Some limitations must be acknowledged. First, although the study population seems to be representative of critically ill COVID-19 patients and the study design resembles clinical routine, which suggests that our findings may be generalizable, further validation in larger and international cohorts is encouraged. Second, possible confounders cannot be ruled out despite adjustment. Third, since we used a real-world clinical practice setting, the sociogeographical context should be considered. For instance, the impact of therapeutic effort limitation on the outcome, SARS-CoV-2 variants and modifications in the treatments during the pandemic cannot be ruled out. Fourth, the role of the miRNAs in the causal pathway of the disease cannot be inferred from our study design, although this knowledge is not a prerequisite for a biomarker. In addition, the role of circulating miRNAs as endocrine genetic signals remains to be fully explored [30]. Fifth, the statistical significance of functional analysis should be treated cautiously since the analysis was based on selected miRNAs. Further studies should explore whether miRNAs participate mechanistically in the pathophysiology of adverse outcomes. Sixth, the current results suggest the need for studies that are more comprehensive and less biased than the panel profiling used here.

To conclude, we validated the use of host miRNA profiles as a source of predictors of fatal outcomes in critically ill COVID-19 patients. In addition, we constructed a 4-blood miRNA classifier that stratifies patients according to their risk of fatal outcome at early stages of ICU admission. This classifier can be integrated into clinical information currently available to improve prognostication in the ICU and therefore to prospectively inform health care management and clinical decision-making.

## Electronic supplementary material

Below is the link to the electronic supplementary material.

Supplementary Material 1

Supplementary Material 2

## Data Availability

The dataset that supports the findings of this study is available from the corresponding author on reasonable request.

## Abbreviations

AUC: area under the ROC curve
CIBER: Centro de Investigación Biomédica en Red
FC: fold change
FDR: false discovery rate
HR: hazard ratio
ICU: Intensive Care Unit
IMV: Invasive Mechanical Ventilation
LASSO: least absolute shrinkage and selection operator
miRNA: microRNA
MSE: mean square error
REDCap: Research Electronic Data Capture
