# Supplementary material for: A blood microRNA classifier for the prediction of ICU mortality in COVID-19 patients: a multicenter validation study
Source: Respir Res. 2023 Jun 17;24:159. doi: 10.1186/s12931-023-02462-x (PMC10276486; doi:10.1186/s12931-023-02462-x)
Supplement: Supplementary file 1 — Supplementary Material 1 [file 12931_2023_2462_MOESM1_ESM.docx]

**A blood microRNA classifier for the prediction of ICU mortality in COVID-19 patients: A multicenter validation study**

David de Gonzalo-Calvo,^1,2^ Marta Molinero,^1,2^ Iván D. Benítez,^1,2^ Manel Perez-Pons,^1,2^ Nadia García-Mateo,^3^ Alicia Ortega,^2,3^ Tamara Postigo,^2,3^ María C. García-Hidalgo,^1,2^ Thalia Belmonte,^1,2^ Carlos Rodríguez-Muñoz,^1,2^ Jessica González,^1,2^ Gerard Torres,^1,2^ Clara Gort-Paniello,^1,2^ Anna Moncusí-Moix,^1,2^ Ángel Estella,^4^ Luis Tamayo Lomas,^2,5^ Amalia Martínez de la Gándara,^6^ Lorenzo Socias,^7^ Yhivian Peñasco,^8^ Maria Del Carmen de la Torre,^2,9^ Elena Bustamante-Munguira,^2,10^ Elena Gallego Curto,^2,11^ Ignacio Martínez Varela,^12^ María Cruz Martin Delgado,^13^ Pablo Vidal-Cortés,^14^ Juan López Messa,^15^ Felipe Pérez-García,^16,17^ Jesús Caballero,^18^ José M. Añón,^2,19^ Ana Loza-Vázquez ,^20^ Nieves Carbonell,^21^ Judith Marin-Corral,^22^ Ruth Noemí Jorge García,^23^ Carmen Barberà,^24^ Adrián Ceccato,^2^ Laia Fernández-Barat,^2,25^ Ricard Ferrer,^2,26^ Dario Garcia-Gasulla,^27^ Jose Ángel Lorente-Balanza,^2,28^ Rosario Menéndez,^2,29^ Ana Motos,^2,25^ Oscar Peñuelas,^2,30^ Jordi Riera,^2,26^ Jesús F. Bermejo-Martin,^2,31^ Antoni Torres,^2,25,32^ Ferran Barbé,^1,2^

1. Translational Research in Respiratory Medicine, University Hospital Arnau de Vilanova and Santa Maria, IRBLleida, Lleida, Spain.

2. CIBER of Respiratory Diseases (CIBERES), Institute of Health Carlos III, Madrid, Spain.

3. Group for Biomedical Research in Sepsis (BioSepsis). Instituto de Investigación Biomédica de Salamanca, (IBSAL), Gerencia Regional de Salud de Castilla y León, Salamanca, Spain.

4. Intensive Care Unit University Hospital of Jerez. Department of Medicine University of Cádiz, INIBiCA, Cádiz, Spain.

5. Critical Care Department, Hospital Universitario Río Hortega de Valladolid, Valladolid, Spain.

6. Department of Intensive Medicine, Hospital Universitario Infanta Leonor, Madrid, Spain.

7. Intensive Care Unit, Hospital Son Llàtzer, Palma de Mallorca, Illes Balears, Spain.

8. Servicio de Medicina Intensiva, Hospital Universitario Marqués de Valdecilla, Santander, Spain.

9. Servei de Medicina Intensiva, Hospital de Mataró (Consorci Sanitari del Maresme), Mataró, Spain.

10. Department of Intensive Care Medicine, Hospital Clínico Universitario Valladolid, Valladolid, Spain.

11. Unidad de Cuidados Intensivos, Hospital Universitario San Pedro de Alcántara, Cáceres, Spain.

12. Critical Care Department, Hospital Universitario Lucus Augusti, Lugo, Spain.

13. Hospital Universitario Torrejón-Universidad Francisco de Vitoria, Madrid, Spain.

14. Intensive Care Unit, Complexo Hospitalario Universitario de Ourense, Ourense, Spain.

15. Complejo Asistencial Universitario de Palencia, Palencia, Spain.

16. Servicio de Microbiología Clínica, Hospital Universitario Príncipe de Asturias – Universidad de Alcalá, Facultad de Medicina, Departamento de Biomedicina y Biotecnología, Madrid, Spain.

17. Centro de Investigación Biomédica en Red en Enfermedades Infecciosas (CIBERINFEC), Instituto de Salud Carlos III, Madrid, Spain.

18. Grup de Recerca Medicina Intensiva, Intensive Care Department Hospital Universitari Arnau de Vilanova, Lleida, Spain.

19. Servicio de Medicina Intensiva. Hospital Universitario La Paz, IdiPAZ, Madrid, Spain.

20. Unidad de Medicina Intensiva, Hospital Universitario Virgen de Valme, Seville, Spain.

21. Intensive Care Unit, Hospital Clínico y Universitario de Valencia, Valencia, Spain.

22. Critical Care Department, Hospital del Mar-IMIM, Barcelona, Spain.

23. Intensive Care Department, Hospital Nuestra Señora de Gracia, Zaragoza, Spain.

24. Intensive Care Department, University Hospital Santa María, IRBLleida, Lleida, Spain.

25. Servei de Pneumologia, Hospital Clinic; Universitat de Barcelona; IDIBAPS, Barcelona, Spain.

26. Intensive Care Department, Vall d’Hebron Hospital Universitari, SODIR Research Group, Vall d’Hebron Institut de Recerca (VHIR), Barcelona, Spain.

27. Barcelona Supercomputing Center (BSC), Barcelona, Spain.

28. Hospital Universitario de Getafe, Madrid, Spain; Dep. of Medicine, Universidad Europea, Madrid, Spain; Dep. of Bioengineering, Universidad Carlos III, Madrid, Spain.

29. Pulmonology Service, University and Polytechnic Hospital La Fe, Valencia, Spain.

30. Hospital Universitario de Getafe, Madrid, Spain

31. Hospital Universitario Río Hortega de Valladolid, Valladolid, Spain; Instituto de Investigación Biomédica de Salamanca (IBSAL), Salamanca, Spain.

32. Institució Catalana de Recerca i Estudis Avançats (ICREA), Barcelona, Spain.

**Corresponding author:**

Ferran Barbé

Translational Research in Respiratory Medicine, University Hospital Arnau de Vilanova and Santa Maria, IRBLleida

Address: Avda. Alcalde Rovira Roure 80 · 25198 Lleida, Spain

Phone: +34 973702491

Email: [febarbe.lleida.ics@gencat.cat](mailto:febarbe.lleida.ics@gencat.cat)

**SUPPLEMENTAL METHODS**

*microRNA quantification*

Blood samples were collected within the first 48 hours of ICU admission into commercially available ethylenediaminetetraacetic acid (EDTA) tubes. The plasma fraction was separated according to standardized operating procedures of each participating hospital and immediately aliquoted, frozen and stored at -80 °C until subsequent analysis. Samples collected at the Hospital Universitario Arnau de Vilanova y Santa María (Lleida, Spain) were obtained with support from IRBLleida Biobank (B.0000682) and “Plataforma Biobancos PT20/00021”. Except for those samples collected at Hospital Universitario Arnau de Vilanova y Santa María (Lleida, Spain), frozen plasma aliquots were received in a centralized manner by the BioSepsis laboratory at the Hospital Universitario Río Hortega (Valladolid, Spain) and then shipped on dry ice to the IRBLleida Biobank (Lleida, Spain). No freeze‒thaw cycles were performed during the experiments.

RNA isolation and miRNA quantification were performed in the same laboratory and under standardized conditions by experienced staff blinded to the clinical data. RNase-free and DNase-free protocols, materials and reagents were used in all procedures. Total RNA was isolated from 180 μL of plasma using a miRNeasy Serum/Plasma Advanced Kit (Qiagen, Hilden, Germany). Synthetic *Caenorhabditis elegans* cel-miR-39-3p (1.6 x 10^8^ copies/μL), lacking sequence homology to human miRNAs, was added as an external reference miRNA. The mixture was supplemented with 1 μL of the RNA Spike-In Kit (UniSp2, 2 fmol/μL; UniSp4, 0.02 fmol/μL; UniSp5, 0.00002 fmol/μL) (Qiagen) to monitor RNA isolation and 1 µg of MS2 bacteriophage RNA (RNA carrier not containing miRNAs) (Roche, Merck, Darmstadt, Germany) to improve extracellular RNA yield. All reagents were spiked into samples during RNA isolation after incubation with the denaturing solution. RNA was eluted into 20 μL of nuclease-free water and stored at -80 °C.

The miRNA panel analyzed was based on published findings of our group [1]. In this discovery study, we identified 16 miRNA candidate biomarkers, from 41 initially evaluated, of ICU mortality, length of ICU stay and/or COVID-19 severity **(Supplemental Table S1; Additional File 2)**. miRNA quantification was performed according to the protocol for the miRCURY LNA Universal RT microRNA PCR System (Qiagen), which offers optimal accuracy and reproducibility [2]. From isolated total RNA, cDNA was synthesized using the miRCURY LNA RT Kit (Qiagen). An additional spike-in UniSp6 (Qiagen) was added to monitor the RT reaction. To avoid the potential inhibitory effect of heparin, heparinase (New England BioLabs, Massachusetts, USA) was added to RT reactions based on previously published data [3]. The RT reactions were performed in a total volume of 10 μL under the following conditions: incubation for 60 minutes at 42 °C, inactivation for 5 minutes at 95 °C and immediate cooling to 4 °C. Then, cDNA was stored at -20 °C. qPCRs were performed in a total volume of 10 μL using miRCURY LNA miRNA Custom Panels (384-well plates), which contained miRNA and spike-in primers (Qiagen). PCR amplification was conducted in an Applied Biosystems QuantStudio™ 7 Flex Real-Time PCR System with the following settings: 95 °C for 2 minutes, followed by 40 cycles of 95 °C for 10 seconds and 56 °C for 1 minute, followed by melting curve analysis. Synthetic UniSp3 was analyzed as an interplate calibrator and qPCR control as described in the kit’s Handbook. Amplification curves were evaluated using QuantStudio Software v1.3 (Thermo Fisher Scientific, Massachusetts, USA). The quantification cycle (Cq) was defined as the fractional cycle number at which the fluorescence exceeded a given threshold. The presence of single products and the absence of primer-dimer were checked by melting curve analysis. In addition, homogeneous efficiencies in RNA extraction, the robustness of cDNA synthesis and the absence of PCR inhibitors were confirmed by analyzing the Cq values of spike-in templates. The method described by Blondal et al. was used to exclude hemolysis contamination [4]. Hemolyzed samples (ΔCq_(miR-23a-3p - miR-451a)_ ≥ 7, n=9) or samples in which miRNA quantification did not pass the quality control (high variability in spike-ins, n=3) were excluded from statistical analysis. Cqs values above 35 cycles were considered undetectable and were censored at the minimum level observed for each miRNA. Relative quantification was performed using the 2^-ΔCq^ method (ΔCq = Cq_miRNA_-Cq_cel-miR-39-3p_). Expression levels were log-transformed for statistical purposes.

*Prediction of microRNA target regulation*

The open-access webserver miRWalk2.0 (version updated in January 2022) was used to predict the interaction of miRNAs with their targets (TargetScan filter, accessed date July 26^th^, 2022) [5]. The analysis was followed by identifying the enriched pathways with the miRWalk2.0 functional enrichment analysis tool. The pathways are presented according to the Reactome, GO and KEGG databases.

**REFERENCES**

1. de Gonzalo-Calvo D, Benítez ID, Pinilla L, Carratalá A, Moncusí-Moix A, Gort-Paniello C, et al. Circulating microRNA profiles predict the severity of COVID-19 in hospitalized patients. Transl Res. 2021;236:147–59.

2. Mestdagh P, Hartmann N, Baeriswyl L, Andreasen D, Bernard N, Chen C, et al. Evaluation of quantitative miRNA expression platforms in the microRNA quality control (miRQC) study. Nat Methods. 2014;11:809–15.

3. Plieskatt JL, Feng Y, Rinaldi G, Mulvenna JP, Bethony JM, Brindley PJ. Circumventing qPCR inhibition to amplify miRNAs in plasma. Biomark Res. 2014;2:1–6.

4. Blondal T, Jensby Nielsen S, Baker A, Andreasen D, Mouritzen P, Wrang Teilum M, et al. Assessing sample and miRNA profile quality in serum and plasma or other biofluids. Methods. 2013;59:S1–6.

5. Sticht C, de La Torre C, Parveen A, Gretz N. miRWalk: An online resource for prediction of microRNA binding sites. PLoS One. 2018;13:e0206239.
