# Supplementary material for: A blood microRNA classifier for the prediction of ICU mortality in COVID-19 patients: a multicenter validation study
Source: Respir Res. 2023 Jun 17;24:159. doi: 10.1186/s12931-023-02462-x (PMC10276486; doi:10.1186/s12931-023-02462-x)
Supplement: Supplementary file 2 — Supplementary Material 2 [file 12931_2023_2462_MOESM2_ESM.docx]

**A blood microRNA classifier for the prediction of ICU mortality in COVID-19 patients: A multicenter validation study**

David de Gonzalo-Calvo,^1,2^ Marta Molinero,^1,2^ Iván D. Benítez,^1,2^ Manel Perez-Pons,^1,2^ Nadia García-Mateo,^3^ Alicia Ortega,^2,3^ Tamara Postigo,^2,3^ María C. García-Hidalgo,^1,2^ Thalia Belmonte,^1,2^ Carlos Rodríguez-Muñoz,^1,2^ Jessica González,^1,2^ Gerard Torres,^1,2^ Clara Gort-Paniello,^1,2^ Anna Moncusí-Moix,^1,2^ Ángel Estella,^4^ Luis Tamayo Lomas,^2,5^ Amalia Martínez de la Gándara,^6^ Lorenzo Socias,^7^ Yhivian Peñasco,^8^ Maria Del Carmen de la Torre,^2,9^ Elena Bustamante-Munguira,^2,10^ Elena Gallego Curto,^2,11^ Ignacio Martínez Varela,^12^ María Cruz Martin Delgado,^13^ Pablo Vidal-Cortés,^14^ Juan López Messa,^15^ Felipe Pérez-García,^16,17^ Jesús Caballero,^18^ José M. Añón,^2,19^ Ana Loza-Vázquez ,^20^ Nieves Carbonell,^21^ Judith Marin-Corral,^22^ Ruth Noemí Jorge García,^23^ Carmen Barberà,^24^ Adrián Ceccato,^2^ Laia Fernández-Barat,^2,25^ Ricard Ferrer,^2,26^ Dario Garcia-Gasulla,^27^ Jose Ángel Lorente-Balanza,^2,28^ Rosario Menéndez,^2,29^ Ana Motos,^2,25^ Oscar Peñuelas,^2,30^ Jordi Riera,^2,26^ Jesús F. Bermejo-Martin,^2,31^ Antoni Torres,^2,25,32^ Ferran Barbé,^1,2^

1. Translational Research in Respiratory Medicine, University Hospital Arnau de Vilanova and Santa Maria, IRBLleida, Lleida, Spain.

2. CIBER of Respiratory Diseases (CIBERES), Institute of Health Carlos III, Madrid, Spain.

3. Group for Biomedical Research in Sepsis (BioSepsis). Instituto de Investigación Biomédica de Salamanca, (IBSAL), Gerencia Regional de Salud de Castilla y León, Salamanca, Spain.

4. Intensive Care Unit University Hospital of Jerez. Department of Medicine University of Cádiz, INIBiCA, Cádiz, Spain.

5. Critical Care Department, Hospital Universitario Río Hortega de Valladolid, Valladolid, Spain.

6. Department of Intensive Medicine, Hospital Universitario Infanta Leonor, Madrid, Spain.

7. Intensive Care Unit, Hospital Son Llàtzer, Palma de Mallorca, Illes Balears, Spain.

8. Servicio de Medicina Intensiva, Hospital Universitario Marqués de Valdecilla, Santander, Spain.

9. Servei de Medicina Intensiva, Hospital de Mataró (Consorci Sanitari del Maresme), Mataró, Spain.

10. Department of Intensive Care Medicine, Hospital Clínico Universitario Valladolid, Valladolid, Spain.

11. Unidad de Cuidados Intensivos, Hospital Universitario San Pedro de Alcántara, Cáceres, Spain.

12. Critical Care Department, Hospital Universitario Lucus Augusti, Lugo, Spain.

13. Hospital Universitario Torrejón-Universidad Francisco de Vitoria, Madrid, Spain.

14. Intensive Care Unit, Complexo Hospitalario Universitario de Ourense, Ourense, Spain.

15. Complejo Asistencial Universitario de Palencia, Palencia, Spain.

16. Servicio de Microbiología Clínica, Hospital Universitario Príncipe de Asturias – Universidad de Alcalá, Facultad de Medicina, Departamento de Biomedicina y Biotecnología, Madrid, Spain.

17. Centro de Investigación Biomédica en Red en Enfermedades Infecciosas (CIBERINFEC), Instituto de Salud Carlos III, Madrid, Spain.

18. Grup de Recerca Medicina Intensiva, Intensive Care Department Hospital Universitari Arnau de Vilanova, Lleida, Spain.

19. Servicio de Medicina Intensiva. Hospital Universitario La Paz, IdiPAZ, Madrid, Spain.

20. Unidad de Medicina Intensiva, Hospital Universitario Virgen de Valme, Seville, Spain.

21. Intensive Care Unit, Hospital Clínico y Universitario de Valencia, Valencia, Spain.

22. Critical Care Department, Hospital del Mar-IMIM, Barcelona, Spain.

23. Intensive Care Department, Hospital Nuestra Señora de Gracia, Zaragoza, Spain.

24. Intensive Care Department, University Hospital Santa María, IRBLleida, Lleida, Spain.

25. Servei de Pneumologia, Hospital Clinic; Universitat de Barcelona; IDIBAPS, Barcelona, Spain.

26. Intensive Care Department, Vall d’Hebron Hospital Universitari, SODIR Research Group, Vall d’Hebron Institut de Recerca (VHIR), Barcelona, Spain.

27. Barcelona Supercomputing Center (BSC), Barcelona, Spain.

28. Hospital Universitario de Getafe, Madrid, Spain; Dep. of Medicine, Universidad Europea, Madrid, Spain; Dep. of Bioengineering, Universidad Carlos III, Madrid, Spain.

29. Pulmonology Service, University and Polytechnic Hospital La Fe, Valencia, Spain.

30. Hospital Universitario de Getafe, Madrid, Spain

31. Hospital Universitario Río Hortega de Valladolid, Valladolid, Spain; Instituto de Investigación Biomédica de Salamanca (IBSAL), Salamanca, Spain.

32. Institució Catalana de Recerca i Estudis Avançats (ICREA), Barcelona, Spain.

**Corresponding author:**

Ferran Barbé

Translational Research in Respiratory Medicine, University Hospital Arnau de Vilanova and Santa Maria, IRBLleida

Address: Avda. Alcalde Rovira Roure 80 · 25198 Lleida, Spain

Phone: +34 973702491

Email: [febarbe.lleida.ics@gencat.cat](mailto:febarbe.lleida.ics@gencat.cat)

**SUPPLEMENTAL MATERIAL**

**Supplemental Tables**

**Supplemental Table S1. microRNA panel and its associations with severity and mortality previously reported in de Gonzalo-Calvo et al. Transl Res, 2021.**

| microRNA ID | Mature sequence | Outcome |
| --- | --- | --- |
| hsa-miR-16-5p | UAGCAGCACGUAAAUAUUGGCG | Severity, in-ICU mortality and duration of ICU stay |
| hsa-miR-27a-3p | UUCACAGUGGCUAAGUUCCGC | Severity |
| hsa-miR-27b-3p | UUCACAGUGGCUAAGUUCUGC | Severity |
| hsa-miR-92a-3p | UAUUGCACUUGUCCCGGCCUGU | Severity, in-ICU mortality and duration of ICU stay |
| hsa-miR-93-5p | CAAAGUGCUGUUCGUGCAGGUAG | ICU stay |
| hsa-miR-98-5p | UGAGGUAGUAAGUUGUAUUGUU | In-ICU mortality |
| hsa-miR-132-3p | UAACAGUCUACAGCCAUGGUCG | In-ICU mortality |
| hsa-miR-148a-3p | UCAGUGCACUACAGAACUUUGU | Severity |
| hsa-miR-150-5p | UCUCCCAACCCUUGUACCAGUG | Severity and duration of ICU stay |
| hsa-miR-192-5p | CUGACCUAUGAAUUGACAGCC | In-ICU mortality |
| hsa-miR-199a-5p | CCCAGUGUUCAGACUACCUGUUC | Severity |
| hsa-miR-214-3p | ACAGCAGGCACAGACAGGCAGU | Duration of ICU stay |
| hsa-miR-323a-3p | CACAUUACACGGUCGACCUCU | In-ICU mortality |
| hsa-miR-451a | AAACCGUUACCAUUACUGAGUU | Severity |
| hsa-miR-486-5p | UCCUGUACUGAGCUGCCCCGAG | Severity |
| hsa-miR-491-5p | AGUGGGGAACCCUUCCAUGAGG | Severity |

ICU: Intensive care unit

| **Supplemental Table S2. Impact of confounding factors on the relationship between microRNAs and all-cause in-ICU mortality.** | | | | | | |
| --- | --- | --- | --- | --- | --- | --- |
|  |  |  |  |  |  |  |
|  | **Unadjusted** | |  |  | **Adjusted** | |
| **microRNA** | **Fold Change** | **p-value** |  | **microRNA** | **Fold Change** | **p-value** |
|  |  |  |  |  |  |  |
| **miR-16-5p** | 0.643 | <0.001 |  | **miR-16-5p** | 0.764 | 0.0170 |
| **miR-92a-3p** | 0.811 | 0.0081 |  | **miR-92a-3p** | 0.875 | 0.1150 |
| **miR-93-5p** | 0.680 | 0.0011 |  | **miR-93-5p** | 0.785 | 0.0520 |
| **miR-98-5p** | 0.593 | 0.0017 |  | **miR-98-5p** | 0.603 | 0.0050 |
| **miR-132-3p** | 0.754 | 0.0387 |  | **miR-132-3p** | 0.752 | 0.0530 |
| **miR-150-5p** | 0.860 | 0.1025 |  | **miR-150-5p** | 0.920 | 0.4010 |
| **miR-192-5p** | 0.695 | 0.0048 |  | **miR-192-5p** | 0.905 | 0.4550 |
| **miR-323a-3p** | 0.640 | 0.0013 |  | **miR-323a-3p** | 0.576 | <0.001 |
| **miR-451a** | 0.675 | 0.0001 |  | **miR-451a** | 0.823 | 0.0530 |
| **miR-486-5p** | 0.738 | 0.0008 |  | **miR-486-5p** | 0.815 | 0.0320 |
|  |  |  |  |  |  |  |
| Adjusted for age, sex, hypertension, chronic pulmonary disease, chronic kidney disease, obesity and diabetes mellitus. | | | | | | |

| **Supplemental Table S3. Association of candidates with all-cause in-ICU mortality.** | | |
| --- | --- | --- |
|  |  |  |
|  | **HR (95% CI)** | **p-value** |
|  |  |  |
| **miR-16-5p** | 1.982 (1.405-2.795) | <0.001 |
| **miR-92a-3p** | 1.371 (0.987-1.904) | 0.06 |
| **miR-93-5p** | 1.742 (1.249-2.430) | 0.001 |
| **miR-98-5p** | 2.614 (1.412-4.839) | 0.002 |
| **miR-132-3p** | 1.536 (1.047-2.251) | 0.028 |
| **miR-192-5p** | 1.851 (1.323-2.589) | <0.001 |
| **miR-323a-3p** | 2.014 (1.352-3.000) | 0.001 |
| **miR-451a** | 1.995 (1.404-2.834) | <0.001 |
| **miR-486-5p** | 1.715 (1.228-2.396) | 0.002 |
|  |  |  |
| HR: Hazard ratio, 95% CI: 95% Confidence interval | | |

| **Supplemental Table S4. Characteristics of patients with COVID-19-related critical excluded and included of the study.** | | | | | |
| --- | --- | --- | --- | --- | --- |
|  | **ALL** | **Excluded patients** | **Included patients** | **p-value** | **Available data** |
|  | N=5067 | N=4576 | N=491 |  |  |
|  |  |  |  |  |  |
| **Sociodemographic characteristics** | | | | | |
| Age (years), median [P25; P75] | 63.0 [54.0;71.0] | 63.0 [54.0;70.0] | 65.0 [56.0;73.0] | <0.001 | 5065 |
| Female, n (%) | 1499 (29.6%) | 1344 (29.4%) | 155 (31.6%) | 0.329 | 5061 |
| Body mass index (kg/cm^2^) , median [P25; P75] | 28.9 [26.0;32.3] | 28.9 [26.0;32.3] | 29.0 [26.1;32.7] | 0.780 | 4447 |
| Smoking history, n (%) |  |  |  | 0.114 | 4334 |
| Former | 1307 (30.2%) | 1162 (30.1%) | 145 (30.7%) |  | |
| Nonsmoker | 2767 (63.8%) | 2478 (64.2%) | 289 (61.2%) |  |  |
| Current | 260 (6.00%) | 222 (5.75%) | 38 (8.05%) |  |  |
|  |  |  |  |  |  |
| **Comorbidities** | | | | | |
| Hypertension, n (%) | 2517 (49.7%) | 2242 (49.0%) | 275 (56.0%) | 0.004 | 5062 |
| Diabetes Mellitus, n (%) | 1246 (24.6%) | 1109 (24.3%) | 137 (27.9%) | 0.084 | 5063 |
| Obesity, n (%) | 1832 (36.3%) | 1648 (36.1%) | 184 (37.5%) | 0.590 | 5052 |
| Cardiovascular disease, n (%) | 618 (13.0%) | 552 (12.9%) | 66 (13.5%) | 0.781 | 4766 |
| Chronic lung disease, n (%) | 491 (9.70%) | 432 (9.45%) | 59 (12.0%) | 0.080 | 5064 |
| Chronic kidney disease, n (%) | 358 (7.07%) | 318 (6.95%) | 40 (8.16%) | 0.368 | 5063 |
|  |  |  |  |  |  |
| **Disease chronology** | | | | | |
| Time since first symptoms to ICU admission (days), median [P25; P75] | 9.00 [7.00;12.0] | 9.00 [7.00;13.0] | 9.00 [7.00;12.0] | 0.097 | 4929 |
| Time since hospital admission to ICU admission (days), median [P25; P75] | 1.00 [0.00;4.00] | 1.00 [0.00;4.00] | 2.00 [0.00;4.00] | 0.470 | 4943 |
| Hospital stay (days), median [P25; P75] | 24.0 [15.0;42.0] | 24.0 [15.0;42.0] | 25.0 [15.0;41.5] | 0.819 | 5063 |
| ICU stay (days), median [P25; P75] | 14.0 [7.00;28.0] | 14.0 [7.00;28.0] | 14.0 [7.00;27.5] | 0.633 | 5031 |
|  |  |  |  |  |  |
| **Blood gases and laboratory parameters at ICU admission** | | | | | |
| Oxygen saturation (%), median [P25; P75] | 94.9 [91.0;97.0] | 95.0 [91.0;97.0] | 94.0 [90.0;97.0] | 0.018 | 4333 |
| PaCO_2_ (mmHg), median [P25; P75] | 39.0 [34.0;47.0] | 39.0 [34.0;47.8] | 38.7 [33.2;45.0] | 0.006 | 4513 |
| pH, median [P25; P75] | 7.41 [7.33;7.46] | 7.41 [7.33;7.46] | 7.41 [7.35;7.45] | 0.633 | 4604 |
| PaO_2_/FiO_2_, median [P25; P75] | 111 [80.0;162] | 111 [80.0;163] | 107 [76.0;148] | 0.020 | 4292 |
| Glucose (mg/dL), median [P25; P75] | 144 [115;194] | 143 [114;193] | 159 [129;207] | <0.001 | 4554 |
| Creatinine (mg/dL), median [P25; P75] | 0.83 [0.67;1.09] | 0.82 [0.66;1.09] | 0.84 [0.70;1.15] | 0.065 | 4964 |
| C-reactive protein (mg/L), median [P25; P75] | 129 [58.0;223] | 130 [58.3;225] | 121 [57.0;216] | 0.427 | 4687 |
| D-dimer (ng/mL), median [P25; P75] | 1070 [554;2749] | 1080 [553;2940] | 1023 [562;1898] | 0.076 | 4364 |
| Leukocyte count (x10^9^/L), median [P25; P75] | 9.25 [6.58;13.0] | 9.20 [6.51;12.9] | 9.90 [7.20;13.8] | 0.001 | 4966 |
| Neutrophil count (x10^9^/L), median [P25; P75] | 7.94 [5.40;11.4] | 7.81 [5.30;11.3] | 8.90 [6.17;12.5] | <0.001 | 4755 |
| Lymphocyte count (x10^9^/L), median [P25; P75] | 0.67 [0.45;0.98] | 0.68 [0.46;0.99] | 0.63 [0.41;0.88] | 0.015 | 4868 |
| Monocyte count (x10^9^/L), median [P25; P75] | 0.37 [0.20;0.56] | 0.37 [0.20;0.55] | 0.40 [0.23;0.56] | 0.135 | 4494 |
| Platelet count (x10^9^/L), median [P25; P75] | 237 [181;311] | 237 [180;312] | 236 [185;307] | 0.776 | 4962 |
| AST (U/L), median [P25; P75] | 45.0 [31.0;71.9] | 46.0 [31.0;72.0] | 42.6 [29.0;68.0] | 0.018 | 3658 |
| ALT (U/L), median [P25; P75] | 40.0 [25.0;66.0] | 40.0 [25.0;65.0] | 41.0 [25.0;67.0] | 0.821 | 4324 |
| Urea (mg/dL), median [P25; P75] | 46.0 [34.0;64.0] | 45.8 [33.0;63.0] | 52.0 [38.0;73.0] | <0.001 | 4069 |
|  |  |  |  |  |  |
| **Severity scores at ICU admission** | | | | | |
| APACHE-II score, median [P25; P75] | 12.0 [9.00;15.0] | 12.0 [9.00;15.0] | 12.0 [9.00;17.0] | 0.022 | 2705 |
| SOFA Score, median [P25; P75] | 5.00 [3.00;7.00] | 5.00 [3.00;7.00] | 5.00 [4.00;7.25] | 0.009 | 3260 |
|  |  |  |  |  |  |
| **Interventions during ICU stay** | | | | | |
| Antibiotics, n (%­­) | 4750 (94.2%) | 4289 (94.2%) | 461 (94.1%) | 0.995 | 5043 |
| Hydroxychloroquine, n (%) | 2310 (45.8%) | 2278 (50.0%) | 32 (6.54%) | <0.001 | 5042 |
| Tocilizumab, n (%) | 2098 (41.6%) | 1973 (43.3%) | 125 (25.6%) | <0.001 | 5042 |
| Corticoids, n (%) | 4410 (87.9%) | 3927 (86.7%) | 483 (98.8%) | <0.001 | 5018 |
| High flow oxygen nasal cannula, n (%) | 2990 (75.6%) | 2643 (75.0%) | 347 (80.5%) | 0.014 | 3955 |
| Non‑invasive positive pressure ventilation, n (%) | 1753 (36.6%) | 1609 (37.4%) | 144 (29.9%) | 0.002 | 4788 |
| Invasive mechanical ventilation, n (%) | 3796 (76.0%) | 3412 (75.8%) | 384 (78.4%) | 0.222 | 4993 |
| Prone positioning, n (%) | 3079 (62.6%) | 2802 (63.3%) | 277 (56.5%) | 0.004 | 4920 |
|  |  |  |  |  |  |
| **Outcome** |  |  |  |  |  |
| All-cause in-ICU mortality, n (%) | 1560 (30.8%) | 1416 (30.9%) | 144 (29.3%) | 0.493 | 5067 |
|  |  |  |  |  |  |
| *Continuous variables are expressed as the median [P25;P75] and categorical variables as n. ALT: alanine aminotransferase; AST: aspartate aminotransferase; FiO_2_: fraction of inspired oxygen; ICU: Intensive care unit; PaCO_2_: carbon dioxide partial pressure; PaO_2_: oxygen partial pressure.* | | | | | |
|  |  |  |  |  |  |

**Supplemental Figures**

**
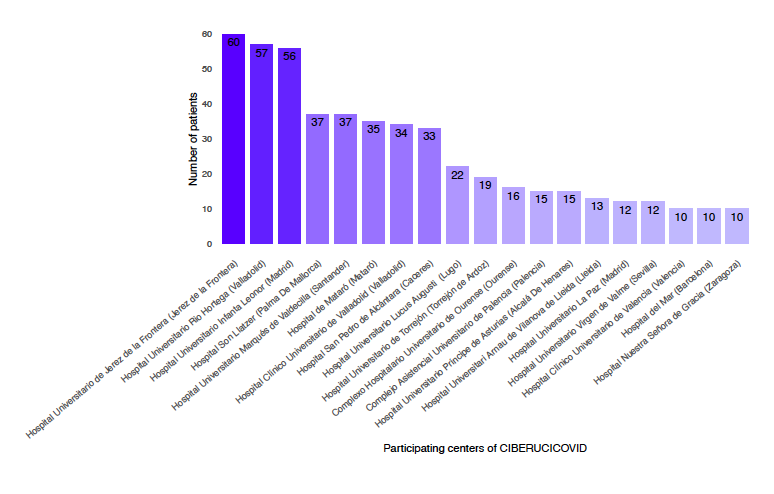
**

**Figure S1. Samples provided by each participating hospital.**


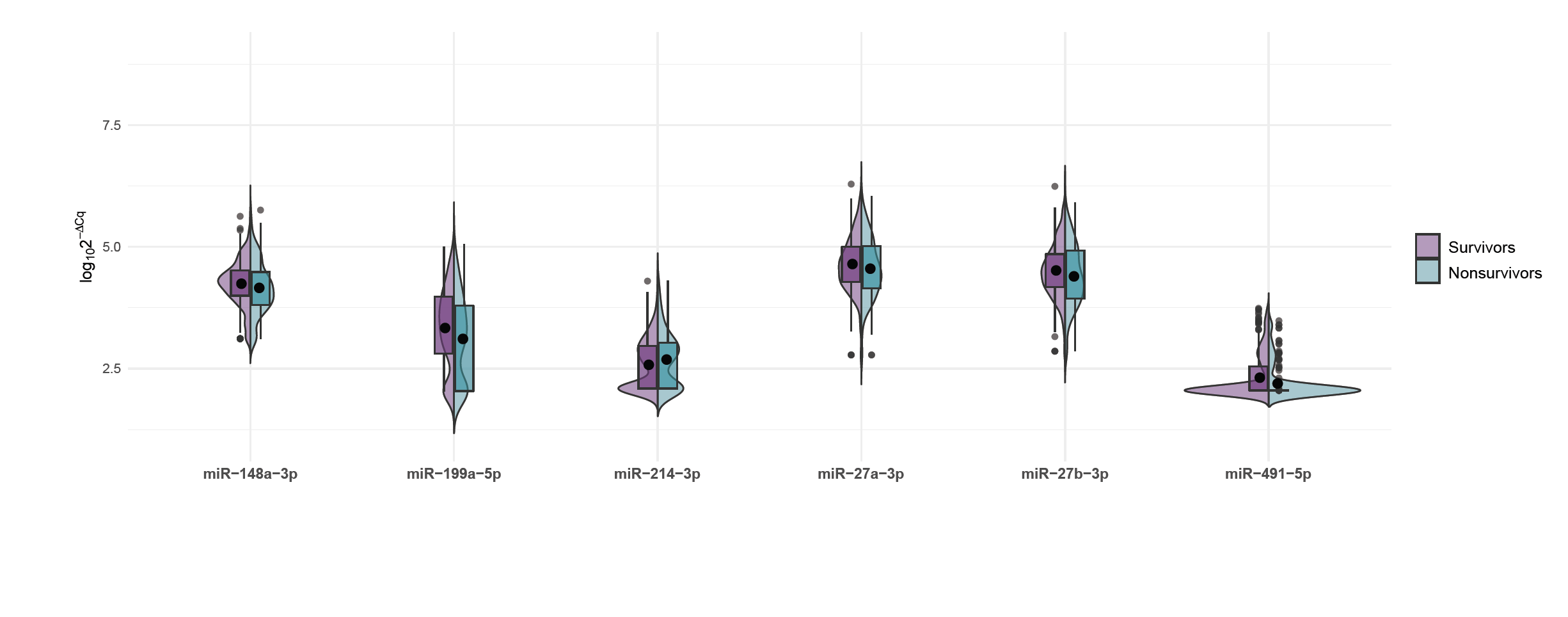


**Figure S2. Nonvalidated microRNAs.** Violin plot comparing nonvalidated microRNA levels between survivors and nonsurvivors. All these candidates showed contradicting results to that observed in a previous study from our group (de Gonzalo-Calvo et al. Transl Res, 2021).


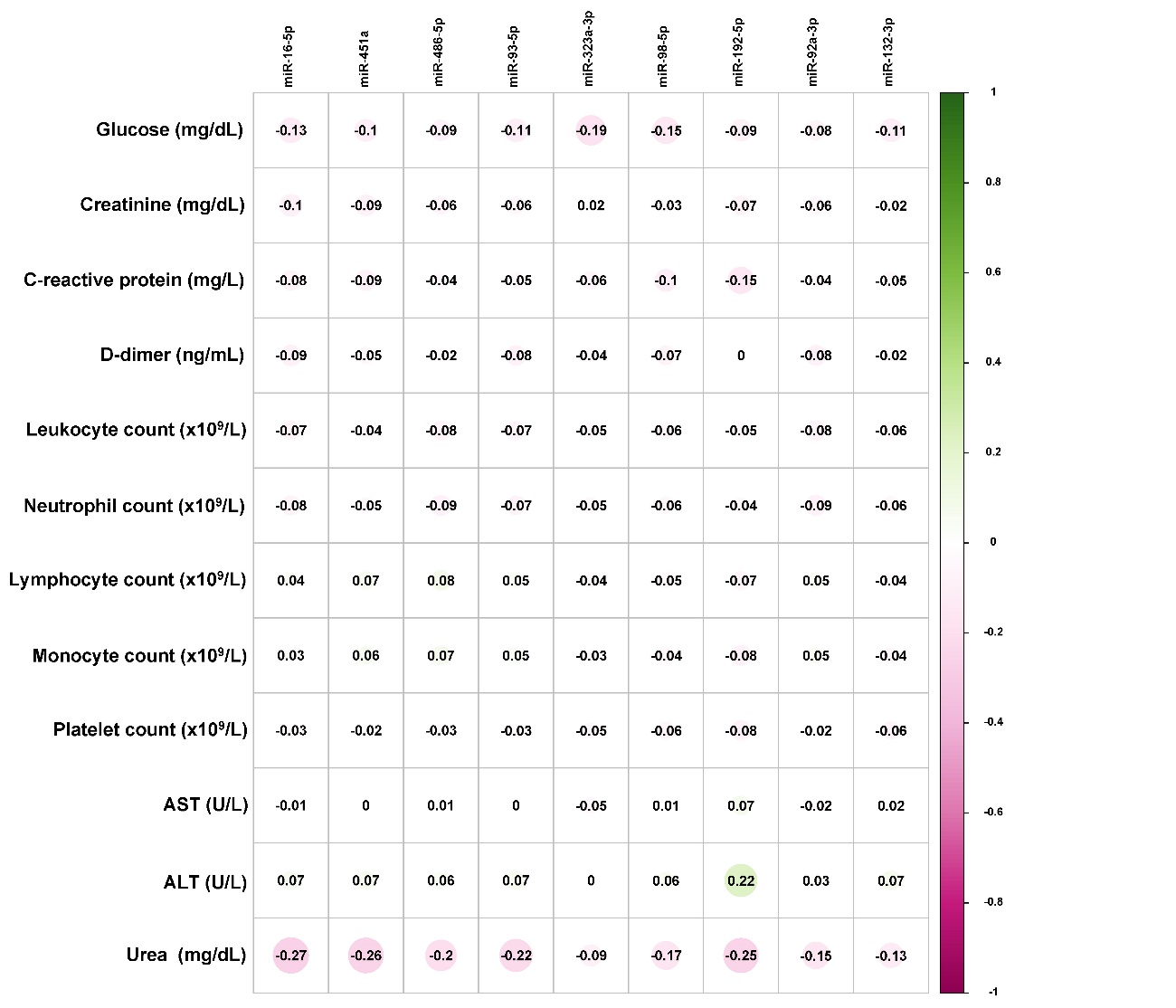


**Figure S3. Correlation between validated microRNAs with blood cell counts and biochemical parameters in the whole population.** Pearson correlation coefficient was used to assess the correlation between continuous variables. ALT: alanine aminotransferase; AST: aspartate aminotransferase.

**
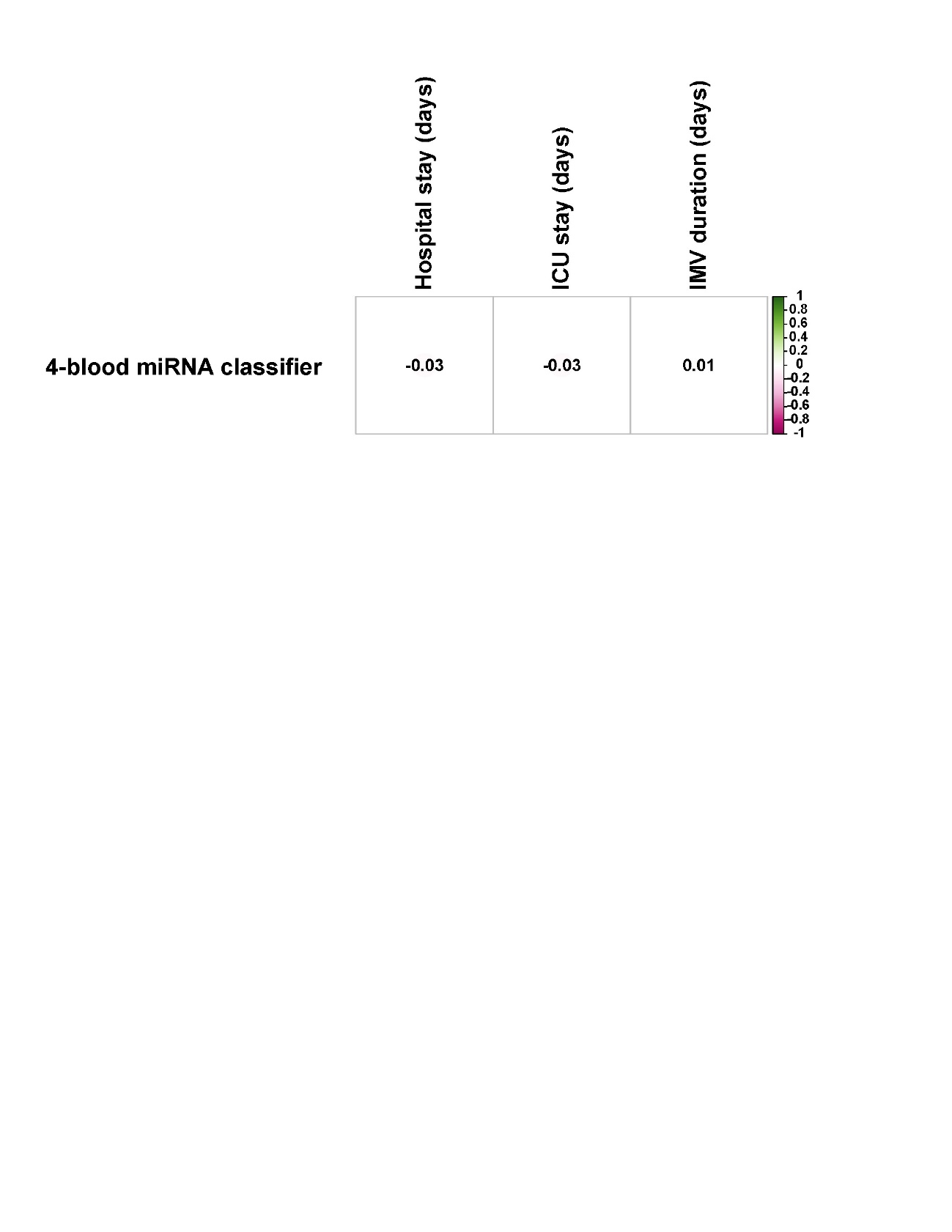
**

**Figure S4. Correlation between the 4-blood microRNA classifier and the duration of hospital stay, ICU stay and invasive mechanical ventilation in** **critically ill survivors.** Pearson correlation coefficient was used to assess the correlation between continuous variables. ICU: Intensive care unit; IMV: invasive mechanical ventilation.

**
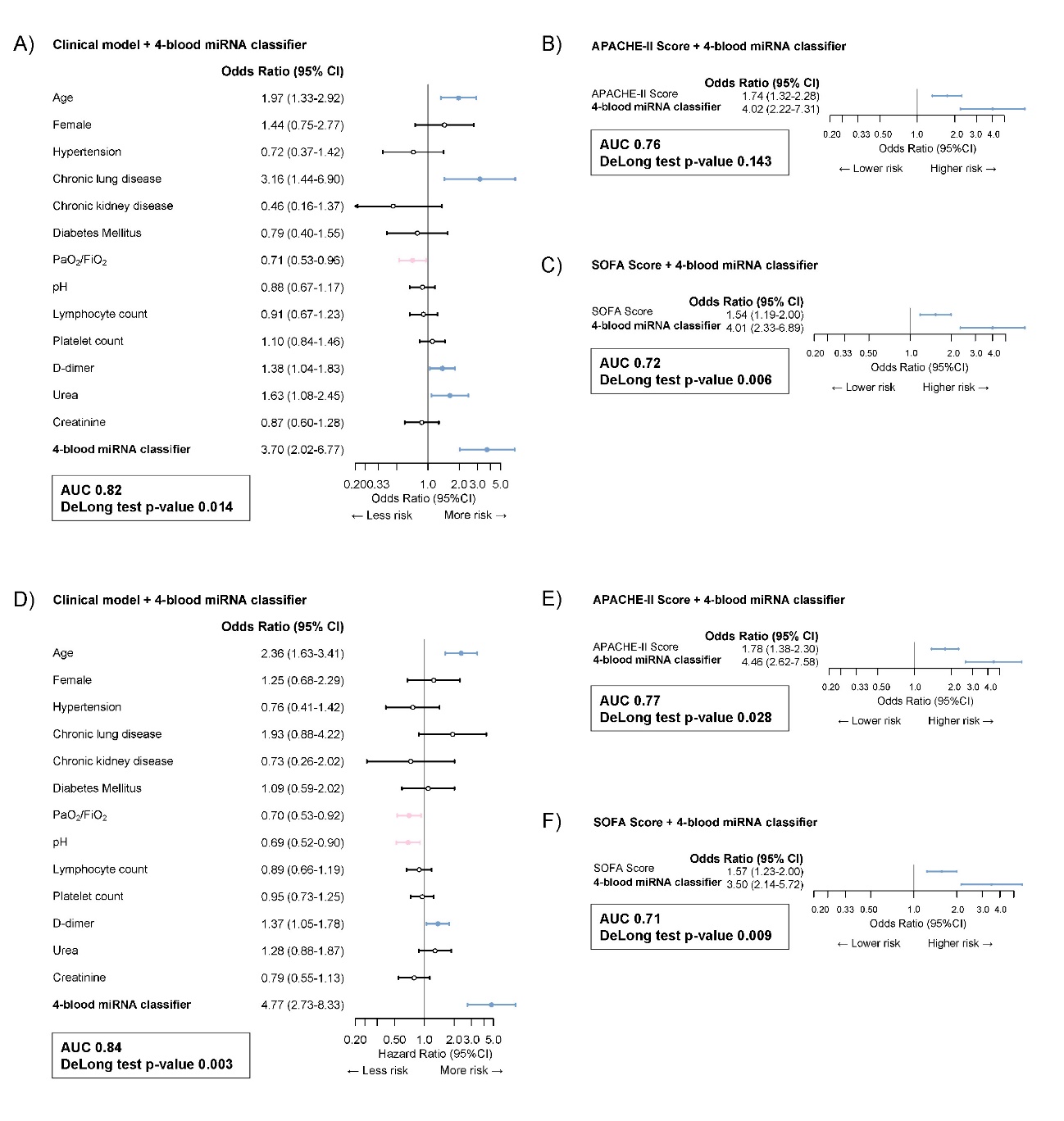
**

**Figure S5. Combination of the 4-blood microRNA classifier with established clinical predictors or contemporaneous prognostic scores for all-cause 28-day mortality (A-C) and all-cause 90-day mortality (D-F).** For all-cause 28-day mortality: A) clinical model (n=377); B) APACHE-II (n=355); C) SOFA (n=379). For all-cause 90-day mortality: D) Clinical model (n=377); E) APACHE-II (n=355); F) SOFA (n=379). The graph displays the Hazard Ratio (HR) and 95% confidence interval (90% CI) for each variable. Cox regression models include the dichotomized levels of the 4-blood microRNA classifier. Hazard Ratio (95%CI) are displayed as 1-SD change for continuous predictor.

**
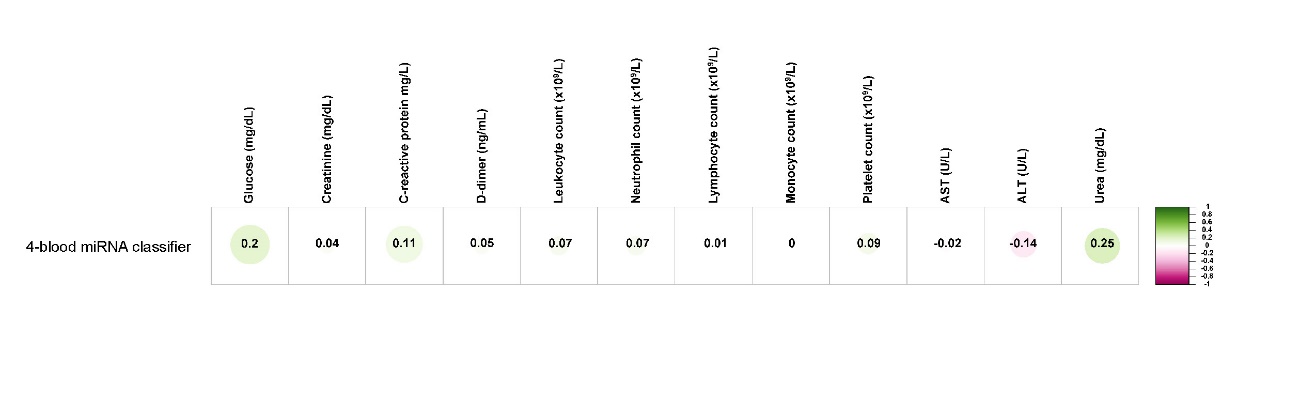
**

**Figure S6. Correlation between the 4-blood miRNA classifier with blood cell counts and biochemical parameters in the whole population.** Pearson correlation coefficient was used to assess the correlation between continuous variables. ALT: alanine aminotransferase; AST: aspartate aminotransferase.
